# Global Gene Expression Analysis of Fission Yeast Mutants Impaired in Ser-2 Phosphorylation of the RNA Pol II Carboxy Terminal Domain

**DOI:** 10.1371/journal.pone.0024694

**Published:** 2011-09-12

**Authors:** Reza Saberianfar, Stephen Cunningham-Dunlop, Jim Karagiannis

**Affiliations:** Department of Biology, University of Western Ontario, London, Ontario, Canada; Texas A&M University, United States of America

## Abstract

In *Schizosaccharomyces pombe* the nuclear-localized Lsk1p-Lsc1p cyclin dependent kinase complex promotes Ser-2 phosphorylation of the heptad repeats found within the RNA pol II carboxy terminal domain (CTD). Here, we first provide evidence supporting the existence of a third previously uncharacterized Ser-2 CTD kinase subunit, Lsg1p. As expected for a component of the complex, Lsg1p localizes to the nucleus, promotes Ser-2 phosphorylation of the CTD, and physically interacts with both Lsk1p and Lsc1p *in vivo*. Interestingly, we also demonstrate that *lsg1Δ* mutants – just like *lsk1Δ* and *lsc1Δ* strains – are compromised in their ability to faithfully and reliably complete cytokinesis. Next, to address whether kinase mediated alterations in CTD phosphorylation might selectively alter the expression of genes with roles in cytokinesis and/or the cytoskeleton, global gene expression profiles were analyzed. Mutants impaired in Ser-2 phosphorylation display little change with respect to the level of transcription of most genes. However, genes affecting cytokinesis – including the actin interacting protein gene, *aip1* – as well as genes with roles in meiosis, are included in a small subset that are differentially regulated. Significantly, genetic analysis of *lsk1Δ aip1Δ* double mutants is consistent with Lsk1p and Aip1p acting in a linear pathway with respect to the regulation of cytokinesis.

## Introduction

In *Schizosaccharomyces pombe*, just as in developmentally complex metazoans, cytokinesis is mediated through the assembly and constriction of a contractile, actomyosin ring [1]. In addition to actin, myosin, and other accessory factors that provide the mechanical force necessary for ring constriction, complex signalling networks also exist to ensure that cytokinesis occurs at the correct location within the cell and at the correct temporal position during the course of the cell cycle [2], [3], [4], [5], [6], [7], [8]. Given the importance of cytokinesis in cellular growth and development, it is not surprising that evidence supporting the existence of a cytokinesis monitoring system has emerged in *S. pombe*. This system has the capacity to both delay G2/M progression and stabilize the actomyosin ring upon perturbation of the division machinery and thus aids in the faithful and reliable execution of fission yeast cytokinesis [9], [10].

Critical components of the monitoring system include the Septation Initiation Network (SIN), the Cdc14p family phosphatase, Clp1p, and the Ser-2 carboxy terminal domain (CTD) kinase, Lsk1p [6], [9], [10], [11], [12], [13], [14]. The SIN is comprised of a complex network of proteins (localizing to the spindle pole body and/or the actomyosin ring) that are required for successful ring constriction following chromosome segregation [6], [12], [13]. Clp1p, on the other hand, is a Cdc14 family phosphatase that plays an important role in SIN activation. While the loss of *clp1* results in only weak cytokinesis defects under normal growth conditions, *clp1Δ* cells display a lethal multi-nucleate phenotype when treated with drugs (Latrunculin A) that perturb the normal function of the actomyosin ring. These defects are in part due to the inability of *clp1Δ* cells to prolong the duration of SIN signaling [9], [10]. Similar to *clp1Δ* cells, *lsk1Δ* mutants appear normal under typical growth conditions, but display striking cytokinesis defects upon perturbation of the cell division machinery. Furthermore – as demonstrated by genetic analysis with hypo- and hyper-active SIN mutants – Lsk1p promotes SIN activation [11].

Rather surprisingly given its role in regulating cytokinesis, *lsk1* encodes a member of a multi-protein complex that specifically phosphorylates the Ser-2 residues present in the twenty-nine heptad repeats (Y_1_S_2_P_3_T_4_S_5_P_6_S_7_) found at the extreme carboxy terminus of the largest subunit of RNA polymerase II, Rpb1p [15], [16], [17]. Lsk1p displays significant sequence similarity to human Cdk9p and budding yeast Ctk1. Human Cdk9p, together with cyclin T, forms the p-TEFb complex, while Ctk1 associates with Ctk2 (cyclin sub-unit) and Ctk3 (gamma sub-unit). Both complexes target Ser-2 residues of the RNA pol II CTD and promote transcript elongation [18], [19].

Importantly, *S. pombe* cells bearing *rpb1* alleles (*rpb1-12XS2ACTD*) that encode 12 copies of a mutant heptad sequence in which alanine has been substituted for serine at the “two” position (in order to mimic the non-phosphorylated state) display cytokinesis phenotypes indistinguishable from *lsk1Δ* strains [14]. These data are consistent with a model in which the CTD is indeed the critical target of Lsk1p with respect to its effects on cytokinesis, and suggests that upstream kinases (or phosphatases) might act through the CTD to selectively control the transcription of certain gene subsets.

In recent years several studies have provided evidence supporting such a hypothesis. For example, work in both *Schizosaccharomyces pombe* and *Saccharomyces cerevisiae* has demonstrated that the impairment of CTD phosphorylation does not necessarily result in generalized defects in transcription or transcriptional control [20], [21], [22]. On the contrary, discrete sub-sets of genes are found to be differentially regulated in such cases. These results demonstrate, at least in principle, that changes in CTD phosphorylation can result in selective as opposed to general alterations in transcription and phenotype.

In this report, we first demonstrate the existence of a third bona fide subunit of the fission yeast Ser-2 CTD kinase complex, Lsg1p (for *l*atrunculin *s*ensitive CTD kinase *g*amma subunit). We also analyze the effects of impaired Ser-2 phosphorylation on the expression of genes with roles in regulating cytokinesis and/or the cytoskeleton. Significantly, strains bearing the *rpb1-12XS2ACTD* mutation display little change with respect to the level of transcription of most genes. However, genes encoding proteins with roles in cytokinesis – including i) the actin interacting protein, Aip1p, ii) the Clp1p interacting protein, Nsk1p, iii) the spindle pole body protein, Cut12p, and iv) Lsk1p itself – are included in a small subset that are differentially regulated. Surprisingly, many genes with roles in the meiotic cell cycle are also significantly affected. This is consistent with recent data demonstrating that Ser-2 phosphorylation is critical for the induction of *ste11* transcription during sexual differentiation [23]. The relevance of this finding within the context of understanding the possible relationship(s) between CTD phosphorylation pattern (i.e. a CTD “code”) and complex phenotypic change is discussed.

## Results

### The annotated *S. pombe* ORF, SPCC4B3.08, defines a putative gamma subunit of the Ser-2 CTD kinase complex

Ser-2 CTD kinase complexes in fungi consist of i) a catalytic kinase subunit, ii) a regulatory cyclin subunit, and iii) a regulatory “gamma” subunit [24]. A BLAST search using a known gamma subunit as query (budding yeast, Ctk3) revealed that the annotated *S. pombe* gene, SPCC4B3.08, was a likely candidate to encode the fission yeast orthologue (Figure S1A). Proteins related to *S. pombe* SPCC4B3.08 at the primary sequence level exist in the fungal kingdom, but not in metazoans (Figure S1B and C). The SPCC4B3.08 gene will hereafter be referred to as *lsg1* for latrunculin sensitive gamma subunit.

### *lsg1* deletion mutants display cytokinesis defects similar to those displayed by *lsk1Δ* and *lsc1Δ* strains

Deletion of the *lsk1* gene in fission yeast confers hyper-sensitivity to low doses of LatA, a drug that inhibits actin polymerization through sequestering actin monomers [25]. At the concentrations used (30–50 times less than that needed to completely depolymerize the actin cytoskeleton) such treatment leads to a Clp1p dependent delay in mitotic entry and the extended activation of the SIN leading to a prolonged cytokinesis-competent state characterized by continuous repair and re-establishment of the actomyosin ring [9], [10]. In the presence of the drug *lsk1Δ* strains are unable to maintain the integrity of the actomyosin ring leading to cytokinesis failure and the generation of inviable, multi-nucleate cells [11].

**Figure 1:**
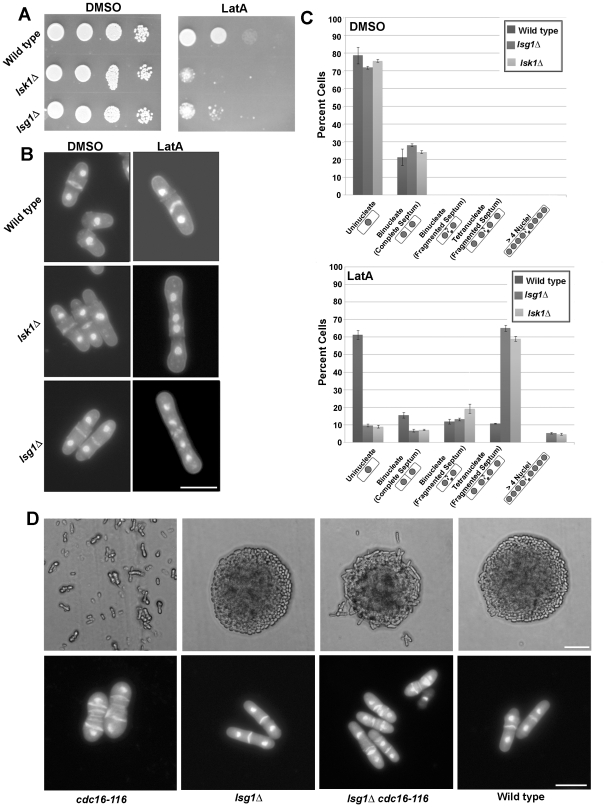
*lsg1* deletion mutants display cytokinesis defects similar to those displayed by *lsk1Δ* strains. (**A**) Ten-fold serial dilutions of logarithmically growing wild-type, *lsg1Δ* and *lsk1Δ* cells were plated onto YES plates containing 0.5 µM LatA or DMSO (solvent control) at 30°C for 3 d. (**B**) Cells of the indicated genotype were grown to mid-log phase at 30°C and then treated with 0.5 µM LatA for 6 h before being fixed and stained with DAPI (nuclei) and aniline blue (cell wall/septa). Bar, 10 µm. (**C**) Quantitation of phenotypes observed for cells treated as in B. Two hundred cells were counted for each genotypic class. Error bars indicate s.d. (n = 3) (D, top panels) Cells of the indicated genotypes were grown at 24°C on YES plates, re-streaked to fresh YES media and then incubated at 36°C for 24 h. Bar, 50 µm (D, bottom panels) Cells of the indicated genotypes were grown to mid-log phase at 24°C and then shifted to 36°C for 4 h. Cells were subsequently fixed and stained with aniline blue (cell wall/septa) and DAPI (nuclei). Bar, 10 µm.

In order to test whether *lsg1* encodes a bona fide member of the CTD complex we first deleted the *lsg1* gene and assayed the mutant for sensitivity to LatA treatment. Ten fold serial dilutions of *lsg1Δ*, *lsk1Δ*, and a wild-type control strain were spotted onto solid media containing LatA (0.5 µM) or DMSO (drug solvent) and the plates incubated for 3 days at 30°C. In contrast to the wild-type control, both *lsg1Δ* and *lsk1Δ* cells displayed a similar hypersensitivity to LatA (Figure 1A).

To further investigate the phenotype of the *lsg1* deletion strain at the microscopic level, *lsg1Δ*, *lsk1Δ* and wild-type cells were grown to mid-log phase at 30°C and treated with LatA for 6 hours. After staining with DAPI and aniline blue (dyes which act as markers for the nucleus and cell wall/septum, respectively) cells were examined by fluorescence microscopy. The cells were classified into five different phenotypic categories: i) uni-nucleate cells, ii) bi-nucleate cells with a morphologically normal septum (i.e. the septum completely bisects the cell), iii) bi-nucleate cells with a fragmented septum (i.e. the septum is non-functional and does not completely bisect the cell), iv) tetra-nucleate cells, and v) cells with greater than four nuclei. As shown in Figures 1B and C, *lsg1Δ*, *lsk1Δ* and wild-type strains did not display any obvious growth defects when grown in the presence of DMSO. In contrast, approximately 60% of *lsg1Δ* and *lsk1Δ* cells displayed a tetra-nucleate phenotype when treated with LatA, whereas only approximately 10% of wild type were tetra-nucleate. Furthermore, cells with greater than four nuclei were only observed in the gene deletion mutants (Figure 1C). The distribution of phenotypes between the various classes was similar in both *lsg1Δ* and *lsk1Δ* strains.

Previous studies have shown that Lsk1p and Lsc1p positively regulate the SIN [11], [14]. For example, the deletion of either *lsk1* or *lsc1* rescues the lethal, multi-septate phenotype conferred by the temperature-sensitive *cdc16-116* allele (this allele results in hyper-activation of the SIN pathway) [26]. To test the effects of the *lsg1Δ* mutation on the SIN, *lsg1Δ cdc16-116* strains were created. As shown in Figure 1D, *cdc16-116* cells could not form colonies and died as single undivided cells at 36°C. In contrast, *lsg1Δ cdc16-116* double mutants could easily form viable colonies. Furthermore, when the cells were examined at the microscopic level, *cdc16–116* mutants showed a strong multi-septate phenotype at 36°C while the *lsg1 cdc16–116* cells exhibited a reduced number of septa and were capable of dividing. This is consistent with a model in which Lsg1p, acts as a positive regulator of the SIN.

### Lsg1p physically associates with Lsk1p and localizes to the nucleus

**Figure 2:**
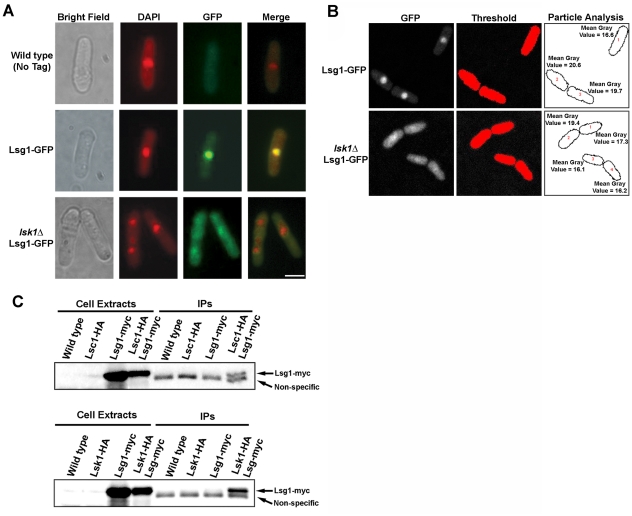
Lsg1p localizes to the nucleus and physically associates with Lsk1p. (**A**) Cells of the indicated genotypes were grown to mid-log phase at 30°C in YES media, fixed, and then stained with DAPI (nuclei) and observed using the DAPI and GFP filter sets. Bar, 5 µm. (**B**) Mean gray level intensity measurements of wild-type (top) or *lsk1Δ* cells (bottom) expressing the Lsg1-GFP fusion protein. Background subtracted images were thresholded and the identified particles quantitatively analyzed using ImageJ. (**C**) Cells expressing the indicated epitope tagged fusion protein were grown to mid-log phase in YES, lysed under native conditions, and subjected to anti-HA immunoprecipitations. Both total lysates (2% input) and immunoprecipitates were resolved by SDS-PAGE and immunoblotted with antibodies specific for the myc epitope. Note the presence of the non-specifically reacting peptide (present in all immunoprecipitates) that acts as a convenient loading control.

As one might expect based on their role in modulating phosphorylation of RNA pol II, both Lsk1p and Lsc1p localize to the nucleus [11], [14]. To test if Lsg1p showed a similar localization, we created a C-terminal *lsg1*-GFP fusion. Analysis of fixed *lsg1*-GFP cells co-stained with DAPI demonstrated that Lsg1-GFP fusion proteins were targetted to the nuclear compartment (Figure 2A). In a complementary experiment, *lsk1Δ* strains expressing GFP-tagged Lsg1p were created to assess the requirement of Lsk1p for the nuclear localization of Lsg1p. This was done since Lsk1p is required for the proper localization of Lsc1p to the nucleus [14]. Analysis of this strain using fluorescence microscopy demonstrated that the Lsg1-GFP signal in *lsk1Δ lsg1-GFP* mutants was spread within the cytoplasm and was not exclusively localized to the nucleus (Figure 2A). To ensure that the overall level of expression of Lsg1-GFP was the same in wild-type and *lsk1Δ* backgrounds, individual cells were analyzed by thresholding background subtracted images and quantitatively analyzing the identified particles (n = 20 for each genotype). The mean gray value (the sum of the gray values of all the pixels in the particle divided by the number of pixels) was not significantly different in wild-type (17.4+/−1.45) vs. *lsk1Δ* mutants (16.5+/−1.69) (Figure 2B). These data demonstrate that Lsk1p is required for the proper localization of Lsg1p to the nucleus, and suggest that it might be important in the intracellular trafficking of the protein.

Next, to determine if Lsg1p physically associates with Lsk1p and Lsc1p *in vivo*, co-immunoprecipitation experiments using myc-tagged *lsg1* alleles were performed. Consistent with the hypothesis that Lsg1p physically interacts with the other two subunits of the complex, Lsg1-myc fusion proteins could be detected with anti-myc antibodies after immuno-precipitation of extracts from *lsg1-myc lsk1-HA* and *lsg1-myc lsc1-HA* strains with anti-HA antibodies (Figure 2C). Taken together, these data strongly suggest that Lsg1p is indeed part of a tri-partite complex with both Lsk1p and Lsc1p.

### Ser-2 phosphorylation is reduced in *lsg1Δ* mutants

**Figure 3:**
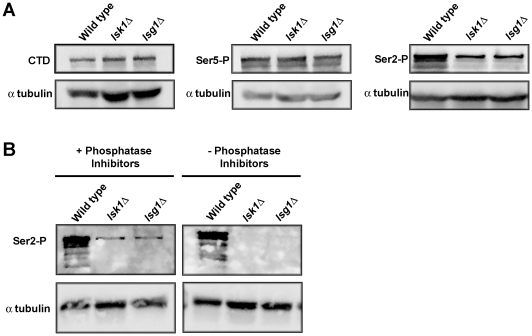
Ser-2 CTD phosphorylation levels are reduced in *lsg1Δ* mutants. (**A**) Cells of the indicated genotypes were grown to mid-log phase in YES. Total lysates were then resolved by SDS-PAGE and immunoblotted with antibodies specific for the unphosphorylated form of the CTD (8WG16), the Ser-5 phosphorylated form of the CTD (H14), the Ser-2 phosphorylated form of the CTD (H5), as well as antibodies specific for alpha-tubulin (loading control). (**B**) Cells of the indicated genotypes were grown to mid-log phase in YES. Total lysates were then prepared using extraction buffer containing or lacking phosphatase inhibitors. Lysates were then resolved by SDS-PAGE and immunoblotted with antibodies specific for the Ser-2 phosphorylated form of the CTD (H5) as well as antibodies specific for alpha-tubulin (loading control).

The Rpb1p CTD can be phosphorylated on both Ser-2 and Ser-5 residues of the heptad repeats [27]. Lsk1p and Lsc1p are required for Ser-2 phosphorylation of the CTD, but do not affect the phosphorylation status of Ser-5 residues [14]. We thus chose to examine phosphoserine-2 levels in *lsg1Δ* mutants relative to wild-type cells. To test if this was the case, western blotting using phosphospecific antibodies was performed to determine the effects of the *lsg1* deletion on the phosphorylation of the CTD. A set of three commercially available phosphospecific antibodies was used: 8WG16 (preferential recognition of unphosphorylated CTD), H14 (preferential recognition of Ser-5 phosphorylated CTD), and H5 (preferential recognition of phospho-Ser-2 and phospho-Ser-2,5) [27], [28]. The level of Ser-2 phosphorylation in *lsg1Δ* and *lsk1Δ* strains was reduced in comparison to wild-type cells. However, there was no significant change in the level of Ser-5 phosphorylation or in the levels of unphosphorylated CTD. Antibodies specific for α-tubulin were used to show that equal amounts of protein were loaded for each sample (Figure 3A).

Interestingly, Karagiannis and Balasubramanian (2007) and others [23] reported that *lsk1Δ* mutations completely abolish Ser-2 phosphorylation. In contrast, the above experiment demonstrated only a reduction in the levels of Ser-2 phosphorylation. It was thus of interest to identify the origin of this discrepancy. Since the original protocols did not include phosphatase inhibitors, the experiment was repeated with and without this treatment. Similar to the previously published work, bands were completely absent from *lsk1Δ* and *lsg1Δ* samples when no phosphatase inhibitors were included in the extraction buffer (Figure 3B). We thus conclude that the inclusion of phosphatase inhibitors in the extraction buffer was the source of the discrepancy in results. Importantly, these data suggest the existence of at least one other kinase capable of phosphorylating Ser-2 residues in the CTD.

### Gene expression analysis

To this point all data were supportive of Lsg1p functioning in a complex with Lsk1p and Lsc1p to phosphorylate Ser-2 residues within the heptad repeats of the Rpb1p subunit of RNA polymerase II. Furthermore, genetic analysis of *lsg1Δ*, *lsk1Δ*, and *lsc1Δ* mutants clearly demonstrated that all three sub-units were important for the reliable execution of cytokinesis. Since mis-regulation of Ser-2 phosphorylation might alter the control of transcription, we decided to analyze the global gene expression profiles of mutants impaired in Ser-2 phosphorylation of the RNA polymerase II CTD. We hypothesized that the transcription of genes involved in cytokinesis or the cytoskeleton might be selectively affected by impairment of Ser-2 phosphorylation. Two strains were used in these studies: a mutant strain bearing the *rpb1-12XS2ACTD* mutation and a control strain of the genotype *rpb1-12XWTCTD*.

The *rpb1* gene in the *rpb1-12XS2ACTD* strain has been mutated so as to encode a total of 12 mutant heptad repeats (Y_1_A_2_P_3_T_4_S_5_P_6_S_7_) in which alanine residues have replaced serine residues at the “two” position. Interestingly, this mutant phenocopies *lsk1Δ*, *lsc1Δ*, and *lsg1Δ* strains in terms of the strain’s inability to complete cytokinesis upon LatA treatment, and in its capacity to rescue the temperature sensitive *cdc16–116* mutation [11], [14]. The *rpb1* gene in the *rpb1-12XWTCTD* strain, on the other hand, encodes a molecule bearing 12 wild-type heptad repeats (Y_1_S_2_P_3_T_4_S_5_P_6_S_7_). This strain displays no obvious growth or morphological phenotypes and does not exhibit any cell cycle or cytokinesis defects. These strains can thus be employed as helpful tools in understanding the effects of the impairment of Ser-2 phosphorylation on cellular physiology and transcription.

To obtain global gene expression profiles, *rpb1-12XCTD* and *rpb1-12XS2ACTD* strains were grown to mid-log phase at 30°C, and then treated with either DMSO (solvent control) or LatA for three hours. Total RNA was then extracted and used in microarray hybridizations using Yeast Genome 2.0 Gene Chips purchased from Affymetrix. Three replicates of each strain (*rpb1-12XCTD* or *rpb1-12XS2A*) under each growth condition (plus or minus LatA) were obtained for a total of 12 samples. After data processing (see Materials and Methods), the twelve samples were grouped according to two parameters: genotype (*rpb1-12XCTD* or *rpb1-12XS2ACTD*) and drug (LatA treated or DMSO treated). In this way, four experimental classes were created: 1) *rpb1-12XCTD,* DMSO treated, 2) *rpb1-12XCTD,* LatA treated, 3) *rpb1-12XS2ACTD*, DMSO treated, and 4) *rpb1-12XS2ACTD*, LatA treated. The complete data set, showing log_2_ normalized intensity values for all genes is included in File S1.

**Figure 4:**
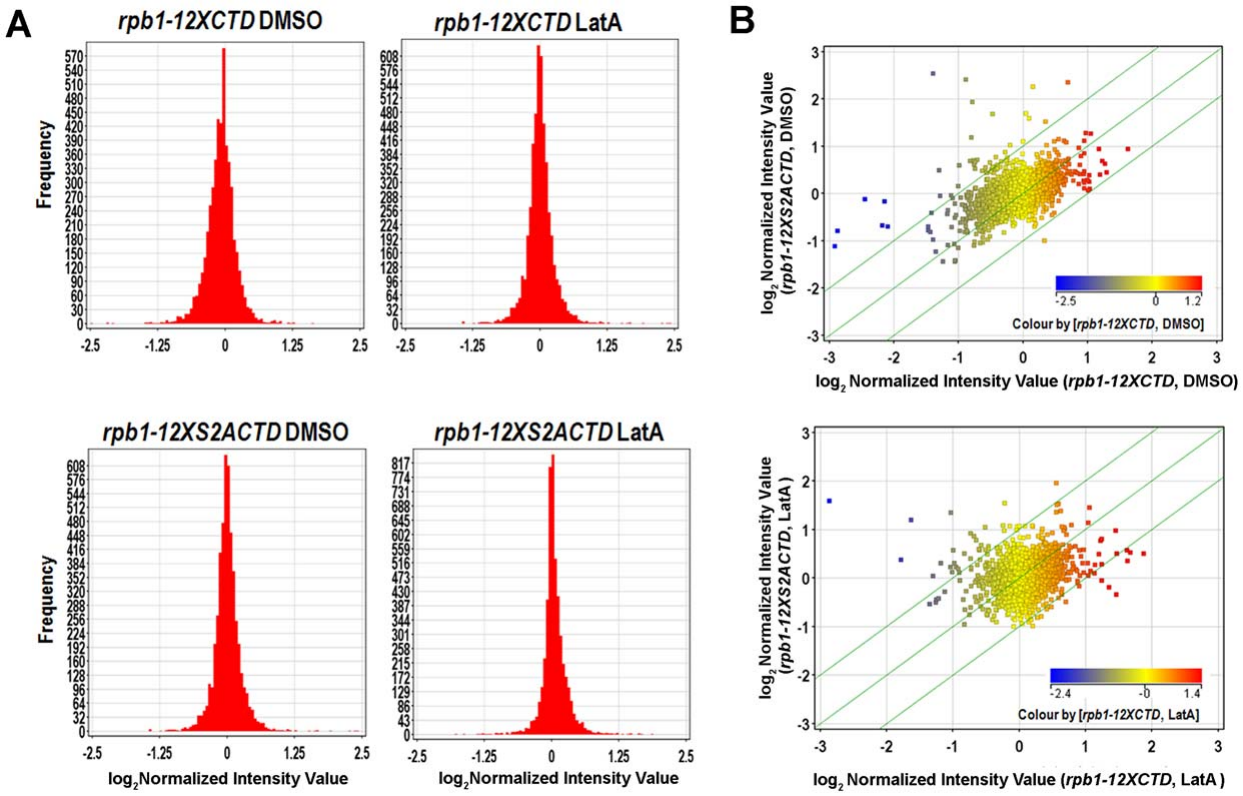
Impairment of Ser-2 phosphorylation results in only modest changes in global gene expression. (**A**) Frequency histograms of genome-wide expression data. Cells of the indicated genotype were grown to mid-log phase at 30°C in YES, and then treated with DMSO or LatA for 3 hours. Total RNA was extracted and used in expression profiling experiments using Affymetrix Yeast 2.0. Genechips. (**B**) Scatter plots comparing gene expression of all *S. pombe* genes (squares) in *rpb1-12XCTD* and *rpb1-12XS2ACTD* strains in the presence of DMSO (top) or LatA (bottom). Diagonal green lines represent the threshold for a 1.5 fold change in expression. Color of squares indicates the level of expression of that gene in DMSO treated *rpb1-12XCTD* cells (top) or LatA treated *rpb1-12XCTD* cells (bottom).

We first examined the gene expression data at the “macro” level to determine if there were any gross differences in the global expression profiles between strains and/or LatA treatment. Frequency distributions of the data revealed only modest changes in expression; all four data sets had similar mean expression levels, standard deviations, as well as similar percentile values (Figure 4A, Table S1). Furthermore, scatterplots comparing *rpb1-12XCTD* and *rpb1-12XS2ACTD* strains treated with either DMSO or LatA (Figure 4B) showed a strong correspondence of expression for the vast bulk of genes. Thus, on a global scale, loss of Ser-2 phosphorylation resulted in only modest changes in the control of gene expression.

### Stress response genes

Next, in order to provide a non-biased assay of the biological veracity of the hybridization data, we examined the core environmental stress response (CESR) genes. The expression levels of these genes is strongly affected by a wide variety of different stresses that include heat shock, cold shock, osmotic stress, oxidative stress, as well as a variety of different DNA damaging agents [29]. The expression of these genes can thus be used as “markers” for exposure to environmental stress. Since one would expect LatA treatment to constitute such a stress, we reasoned that the expression levels of the CESR genes could be used as an informative control to ensure that the expression arrays were providing biologically meaningful data.

**Figure 5:**
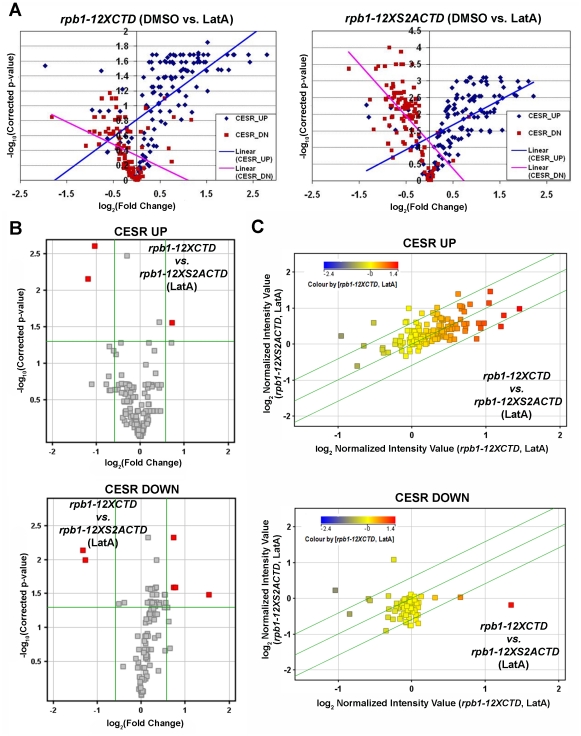
*rpb1-12XS2ACTD* mutants do not exhibit a generalized defect in mounting a proper transcriptional response to stress. (**A**) Volcano plot analysis of the expression of the Core Environmental Stress Response (CESR) genes in *rpb1-12XCTD* strains treated with LatA (left) and *rpb1-12XS2ACTD* strains treated with LatA (right). Blue diamonds represent CESR genes normally up-regulated in response to multiple stresses. Red squares represent CESR genes normally down-regulated in response to multiple stresses. (**B**) Volcano plot analysis of the expression of CESR genes (Up-regulated, left; Down-regulated, right). Plots compare *rpb1-12XCTD* vs. *rpb1-12XS2ACTD* strains treated with LatA. Horizontal green line represents p-value of 0.05. Vertical green lines represent threshold for a 1.5 fold change in expression. (**C**) Scatter plots comparing gene expression of CESR UP genes (top) and CESR DOWN genes (bottom) between genotype in the presence LatA. Diagonal green lines represent the threshold for a 1.5 fold change in expression. Color of squares indicates the level of expression of that gene in LatA treated *rpb1-12XCTD* cells.

To analyze the data, the CESR gene set was divided into two individual groups, the CESR-Up group (comprised of genes that are up-regulated upon exposure to environmental stresses) and the CESR-Down group (comprised of genes that are down-regulated upon exposure to environmental stress). The expression levels of these two groups of genes in DMSO and LatA treated *rpb1-12XCTD* cells, as well as DMSO and LatA treated *rpb1-12XS2ACTD* cells, were compared using volcano plot (a scatter-plot of the -log_10_-transformed p-values from gene-specific t*-*tests vs. the log_2_ fold change). As seen in Figure 5A the CESR-Up genes (blue) are generally up-regulated in response to LatA treatment, while the CESR-Down genes (red) are generally down-regulated in both genetic backgrounds. Furthermore, regression lines fitted to the CESR-Up and CESR-Down genes, result in lines of positive and negative slope respectively, as expected. These results indicated that the microarray data was providing accurate and biologically relevant expression data.

Since it was conceivable that the LatA hyper-sensitivity observed in *rpb1*-*12XS2ACTD* strains was due to defects in mounting an appropriate transcriptional response to stress, we also compared CESR gene expression between genotype. As expected, no significant differences in CESR gene expression were observed between DMSO treated *rpb1-12XCTD* and *rpb1-12XS2ACTD* strains (data not shown). Furthermore, in response to LatA treatment, only three (out of 136) CESR-UP genes, and six (out of 104) CESR-DOWN genes were differentially regulated between genotype (Figure 5B an C). Lastly, when we expanded the analysis to include any gene annotated by the Gene Ontology Consortium as having a role in the cellular response to stress, we reached similar conclusions; only 8 out of 561 genes in the set were differentially regulated between genotype upon LatA treatment while no genes in the set were differentially regulated upon DMSO treatment (Figure S2). These results strongly suggest that the LatA sensitivity conferred by *rpb1-12XS2ACTD* mutations does not not stem from any gross failings in the control of expression of stress response genes.

### Cytokinesis genes

**Figure 6:**
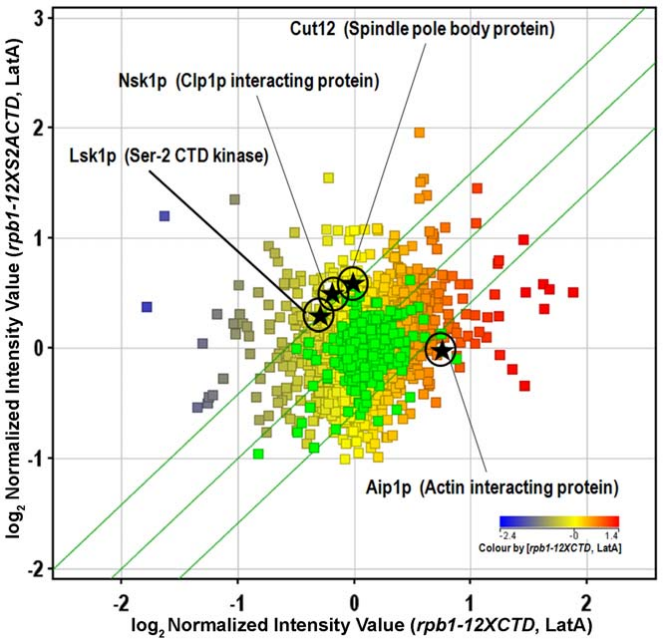
Expression levels of only a small subset of cytokinesis related genes are significantly altered by impairment of Ser-2 phosphorylation. Cells of the indicated genotypes were grown to mid-log phase at 30°C in YES, and then treated with DMSO or LatA for 3 hours. Total RNA was extracted and used in expression profiling using Affymetrix Yeast 2.0. Genechips. Graph shows scatter plot analysis comparing gene expression of all *S. pombe* genes (squares) in *rpb1-12XCTD* and *rpb1-12XS2ACTD* strains in the presence of LatA. Genes with known roles in cytokinesis are shown in green. Significantly affected cytokinesis genes are shown as black stars. Green lines represent the threshold for a 1.5 fold change in transcript levels. Color of non-green squares indicates the level of expression of that gene in LatA treated *rpb1-12XCTD* cells.

Since *rpb1-12XS2ACTD* strains display defects related to cytokinesis we next analyzed genes annotated as having roles in cytokinesis or in controlling the cytoskeleton (a total set of 333 genes) (see File S1 for a complete list). To identify differentially expressed genes we filtered the data using volcano plot analysis to screen for statistically significant changes (p-value<0.05) greater than 1.5 fold in magnitude. When DMSO treated *rpb1-12XCTD* and *rpb1-12XS2ACTD* strains were compared – conditions where the two strains show no obvious phenotypic differences – there were no cytokinesis genes found to be differentially regulated. When LatA treated *rpb1-12XCTD* and *rpb1-12XS2ACTD* strains were compared only 4 out of 333 genes were differentially regulated (Figure 6). While this low number was somewhat surprising, the identities of the genes were highly notable. The first of the three up-regulated genes was *lsk1* itself, suggesting the existence of positive feedback between low phosphoserine-2 levels and the control of *lsk1* expression. The second of the up-regulated genes, *nsk1*, encodes a known physical interactor of Clp1p, a previously characterized regulator of the cytokinesis monitoring system [9], [10]. The third of the up-regulated genes, *cut12*, encodes a spindle pole body protein with known roles in regulating mitosis, cytokinesis, and the SIN [30], [31], [32]. Intriguingly, the only down-regulated gene in the set, *aip1*, encodes an actin interacting protein whose human orthologue has a known role in preventing cytokinesis failure in HeLa cells [33].

### Genetic Analysis

**Figure 7:**
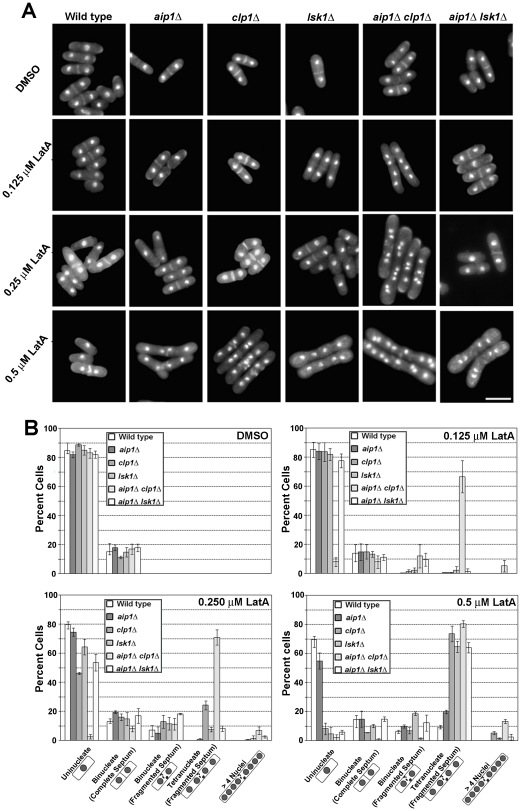
Epistasis analysis of *lsk1Δ aip1Δ*and *clp1Δ aip1Δ* double mutants. (**A**) Strains of the indicated genotype were grown to mid-log phase in YES at 30°C and treated with LatA or DMSO (solvent control) for 6 h. Cells were fixed and stained with DAPI (DNA) and aniline blue (cell wall/septum). Bar, 10 µm. (**B**) Quantitation of phenotypes observed for cells treated as in A. Two hundred cells were counted for each genotypic class. Error bars indicate s.d. (n = 3).

To explore whether any of these differentially regulated genes might be relevant to the LatA sensitivity of *rpb1-12XS2ACTD* strains, we chose to focus on *aip1* (analysis of *nsk1* and *cut12* will be presented elsewhere). To examine the role of Aip1p we analyzed *aip1Δ*, *aip1Δ clp1Δ* and *aip1Δ lsk1Δ* mutants and assayed their LatA sensitivity. *aip1Δ clp1Δ* were analyzed since loss of *clp1* exacerbates the cell division defects of a variety of diverse cytokinesis mutants. Moreover, mutants with weak cytokinesis defects often display strong phenotypes in *clp1Δ* backgrounds. While *aip1Δ* single mutants showed only modest defects, *aip1Δ clp1Δ* double mutants were exquisitely sensitive to LatA treatment. Even at very low concentrations – where both single mutants and the wild-type were insensitive – double mutants were unable to complete cytokinesis and accumulated multiple nuclei (Figure 7). Also of significance is the observation that no such synthetic interaction was observed in *aip1Δ lsk1Δ* (Figure 7) or *rpb1-12XS2ACTD aip1Δ* (data not shown) strains i.e. the double mutants were no more sensitive than *lsk1Δ* or *rpb1-12XS2ACTD* single mutants. These results demonstrate that Aip1p does indeed influence sensitivity to LatA in fission yeast. Furthermore, the fact that loss of Aip1p increased sensitivity in *clp1Δ* strains, but had no effect in *lsk1Δ* strains, is consistent with *aip1* acting in a parallel pathway to *clp1* and in a linear pathway with *lsk1*. These data are thus consistent with a model in which the control of *aip1* transcription is part of a transcriptional “program” affected by Lsk1p mediated phosphorylation of the CTD.

### Meiosis genes

We next examined the global set for genes significantly affected by genotype. Upon LatA treatment we found that only 152 out of 4875 genes (approximately 3%) were differentially regulated (fold-change >1.5, p<0.05) when comparing *rpb1-12XCTD* and *rpb1-12XS2ACTD* strains (see File S1 for a complete list). In this set were a diverse set of genes involved in cellular processes including metabolism, membrane transport, and cell wall architecture. Despite the apparent diversity, a clear pattern in these genes was noted upon careful analysis of the data. Of the gene set, 47 (approximately 31%) were found to be part of the transcriptional program initiated upon entry into the meiotic cell cycle. By cross referencing to data [34] made freely available from the Bahler lab(http://www.bahlerlab.info/projects/sexualdifferentiation/meiosis/) we found that 41 of these genes were induced upon entry into the meiotic cycle, whereas 6 were repressed (Figure S3). Consistent with this result, Ser-2 phosphorylation was recently shown to be important for the induction of *ste11* transcription during sexual differentiation. Moreover, homothallic *lsk1* gene deletion mutants were also found to display meiotic defects [23]. Taking all data together, these results are consistent with a model in which the modulation of CTD phosphorylation results in selective changes in a limited set of genes and in discrete phenotypic alterations.

## Discussion

In this report, we identify the gene product of the *S. pombe* ORF, SPCC4B3.08, as a bona fide member of the fission yeast Ser-2 CTD kinase complex. This conclusion is based both on biochemical evidence demonstrating physical interaction between Lsg1p and the Lsk1p and Lsc1p sub-units (Figure 2C), together with genetic evidence showing that *lsg1* gene deletion mutants exhibit cytokinesis phenotypes indistinguishable from *lsk1Δ* strains (Figure 1). We also demonstrate that Lsg1p localizes to the same intracellular compartment as Lsk1p, and moreover that this intracellular localization is dependent on Lsk1p (Figure 2A and B). This last observation highlights the interdependence of the Ser-2 CTD kinase complex and suggests that Lsg1p is targeted to the nucleus through its association with Lsk1p. Alternatively, Lsk1p mediated phosphorylation of Lsg1p might be important for intracellular trafficking of the Lsg1p protein independently of its association with Lsk1p.

While clearly required for the Ser-2 specific phosphorylation activity of the kinase complex (Figure 3), the mechanism by which “gamma” subunits in general, and Lsg1p in particular, affect the catalytic kinase component of the complex remains poorly understood. While the stability of budding yeast Ctk3 is known to be regulated through the presence of a PEST domain [35], Lsg1p does not possess an orthologous sequence (Figure S1). This observation thus precludes the modulation of Lsg1p stability through a PEST domain as a possible means of regulatory control over Ser-2 CTD kinase activity. In other tri-partite CDK complexes such as the Mat1-cyclin H-CDK 7 complex, the non-cyclin regulatory sub-unit is known to stabilize the cyclin and kinase components and thus to promote the formation of a catalytically active CDK [36]. Regardless of the molecular mechanism(s) regulating the complex, it is interesting to speculate as to whether the Lsg1p and Lsc1p sub-units might be sensitive to distinct cellular signals. In this way the control of CTD phosphorylation could be finely tuned to integrate information from a wide variety of diverse signalling pathways.

When considering such models it is intriguing to note the variety of potential phosphorylation sites available to act as substrates for regulatory kinases and/or phosphatases (29 copies of the heptad sequence exist in fission yeast, whereas 52 repeats exist in humans). Thus, differential phosphorylation of the CTD may define specific “codes” which can be “read” by CTD binding proteins to influence transcriptional control [15], [16], [37], [38]. This potential complexity has led some to speculate that the CTD is crucial for the intricate and selective regulation of genetic networks in eukaryotes. For example, Stiller and Hall (2002) have hypothesized that acquired CTD/protein interactions have been crucial in the evolution of complex patterns of gene expression characterizing developmentally complex eukaryotes. The importance of the CTD is also borne out by the observation that it is required for viability, but not for basal transcriptional activity in vitro [16], [17], [38], [39]. This key observation strongly suggests that, while the CTD is not catalytically essential, it must perform other crucial functions within eukaryotes [15], [37].

In addition to these arguments, recent empirical evidence has also appeared suggesting complex roles for the CTD in the regulation of discrete genetic pathways within eukaryotic cells. First, work in *S. pombe* has clearly shown that alterations in CTD phosphorylation do not result in generalized defects in the overall level of transcription. For example, Lee et al., (2005) demonstrate that the reduction of Ser-5 phosphorylation (through mutation of the *cdk7* orthologue, *mcs6*) leads to the reduction of a sub-set of transcripts involved in the regulation of septum dissolution at the end of cytokinesis. Furthermore, temperature-sensitive *mcs6* mutants die with a phenotype characteristic of mutants that are unable to fully degrade the septum after cytokinesis.

Even more intriguing is the observation that the phosphorylation status of the CTD can be affected by environmental conditions. For example, the level of Ser-2 phosphorylation increases both upon heat shock, and during the diauxic shift in *Saccharomyces cerevisiae*. In addition, phosphoserine-2 levels are also seen to increase upon exposure to DNA damaging agents in a manner dependent on the Ser-2 CTD kinase, Ctk1p [21], [22]. Interestingly, *ctk1Δ* mutants are sensitive to these very same DNA damaging agents. Moreover, genes involved in DNA repair are amongst the *ctk1* dependent genes that are transcriptionally altered by exposure to DNA damage. These observations suggest that CTD phosphorylation plays a role in the transcriptional control of genes involved in the DNA damage response.

In this report we provide further support for a model in which the CTD influences the selective modulation of gene expression. We clearly show that impairment of Ser-2 phosphorylation does not lead to gross changes in transcriptional profile. Both *rpb1-12XCTD* and *rpb1-12XS2ACTD* strains display a similar distribution of gene expression levels (Figure 4A) as well as similar mean expression and percentile rankings (Table S1). In addition, scatterplots show a strong correspondence of expression for the vast bulk of genes (Figure 4B). Indeed, only 152 out of 4875 *S. pombe* genes (and four out of 333 “cytokinesis” genes) were found to be differentially regulated between genotype upon LatA treatment.

While such a small number was somewhat unexpected, the identities of the cytokinesis related genes were striking. The first of the three up-regulated genes was *lsk1* itself. While not informative with respect to the cytokinesis phenotype, the identification of Lsk1p is significant since it suggests the existence of positive feedback between low phosphoserine-2 levels and the control of *lsk1* expression. That is to say, it is conceivable that low phosphoserine-2 levels in the *rpb1-12XS2ACTD* strain might be sensed by the cell and trigger increased *lsk1* expression to compensate. The second of the up-regulated genes, *nsk1*, encodes a Clp1p interacting protein. Given that Clp1p is a critical regulator of the cytokinesis monitoring system in *S. pombe*, it is plausible that changes in *nsk1* expression could be related to the LatA sensitivity of *rpb1-12XS2ACTD* strains. The third of the up-regulated genes, *cut12*, encodes a spindle pole body protein with known roles in regulating the Plo1p kinase which in turn acts as a positive regulator of the SIN [30], [31], [32]. The only down-regulated gene in the set, *aip1*, encodes an actin interacting protein. Intriguingly, when human orthologues of this gene are knocked down in HeLa cells, both cytokinesis failure and an accumulation of multinucleate cells are observed [33].

While we favor a model in which the subset of differentially regulated genes represents the output of a transcriptional “program” – where multiple genes contribute to phenotype – we were able to provide evidence that one of the identified cytokinesis genes, *aip1*, does indeed play a role in promoting the successful completion of cytokinesis (Figure 7). While no severe defects were detected in otherwise wild-type backgrounds, *aip1* gene deletion mutants displayed strong synthetic interactions with *clp1Δ* mutants. This was not surprising since weak cytokinesis mutants have been observed to display strong phenotypes when placed in backgrounds where the cytokinesis monitoring system is non-functional [9], [10]. Moreover, whereas the LatA hypersensitivity of *clp1Δ* mutants was exacerbated by the *aip1* deletion, the LatA sensitivity of *lsk1Δ* cells remained unaffected in *aip1Δ* backgrounds. This result suggests that Lsk1p and Aip1p act in a linear pathway and would thus be consistent with a model in which Lsk1p mediated changes in CTD phosphorylation selectively affects the transcription of genes affecting LatA hyper-sensitivity.

Lastly, a close inspection of the data also revealed the selective mis-regulation of genes involved in meiosis. Of the differentially regulated genes, 47 (approximately 31%) were found to be trascriptionally up- or down-regulated upon entry into the meiotic cell cycle (Figure S3). Remarkably, this is consistent with recent data showing a requirement for Lsk1p in the transcriptional program initiated upon sexual differentiation [23]. These results bring up the question of whether the cytokinesis phenotype might be related to defects in the meiotic transcriptional program. In this respect it is interesting to note that the disruption of components of the MAPK signalling pathway (Ste20p, Ste11p, Ste7p) leading to the activation of the budding yeast Ste12p transcription factor (the functional equivalent to fission yeast Ste11p), as well as loss of function mutations in budding yeast *ste12* itself, cause defects in the expression of genes involved in cell wall integrity during vegetative growth [40]. This result sets a precedent in establishing a role for meiotic specific regulators during the vegetative cell cycle, and moreover, brings to mind the possibility that *ste11* dependent genes might play a morphogenetic role during cytokinesis. In any event, taking all results together, our data are consistent with a model in which alterations in CTD phosphorylation result, not in drastic global changes in transcription, but rather in selective changes in a limited set of genes resulting in discrete phenotypic changes. Regulatory modules that function through the fine-tuned modulation of CTD phosphorylation might thus represent a previously underappreciated mechanism of genetic control in eukaryotes.

## Materials and Methods

### Yeast Methods

All *Schizosaccharomyces pombe* strains used in this study (Table S2) were either obtained from the Karagiannis lab collection, created during the course of this study, or in the case of the *aip1* gene deletion strain, purchased from Bioneer Corporation (Alameda, CA). *Schizosaccharomyces pombe* cells were cultured in either YES or Edinburgh Minimal Media (EMM) (Forsburg and Rhind 2006) with the appropriate supplements (Adenine, Histidine, Leucine, or Uracil). Liquid cultures were grown with shaking (200 rpm) at 30°C. Genetic crosses were performed using standard methods [41]. In experiments involving Latrunculin treatment, *S. pombe* cells were grown to mid log phase (O.D. 0.2) and treated with 0.2-0.5 µM of Latrunculin A (Enzo Life Sciences International, Plymouth Meeting, Pennsylvania) dissolved in DMSO. Cells were grown at 30°C with shaking at 200 rpm for 3-6 hrs, before being fixed. Plasmid vectors were transformed into *S. pombe* using the lithium acetate protocol according to Forsburg and Rhind [41].

### Cloning Methods

To create the *lsg1* gene deletion mutant, two separate fragments corresponding to sequences upstream and downstream of the *lsg1* open reading frame (SPCC4B3.08) were PCR amplified from wild type genomic DNA using primers RS1, RS2, RS3 and RS4 (Table S3). The 226 bp fragment generated using RS1 and RS2 primers was cloned upstream of the *ura4^+^* gene (used as a selectable marker) in the pSKURA4 plasmid using *Kpn*I and *Xho*I restriction sites. The 310 bp fragment generated using RS3 and RS4 primers was cloned downstream of the *ura4^+^* using *Xba*I and *Sac*I restriction sites. Digestion with *Kpn*I and *Sac*I liberated a linear dsDNA fragment composed of the 226 bp upstream fragment, the *ura4+* selectable marker, and finally the 310 bp downstream fragment. This linear dsDNA fragment was then used to transform *S. pombe* strain MBY1343. Ura*^+^* transformants were isolated and subject to colony PCR using primers RS10, RS11, ME12 and ME28 to identify clones in which the linear dsDNA fragment had replaced the endogenous *lsg1* gene via homologous recombination.

To create the Lsg1-GFP integrant strain, a C-terminal fragment of the *lsg1* gene was PCR amplified from genomic DNA using primers RS5 and RS6 (Table S3). The fragment was then cloned upstream and in frame to the GFP gene located in the pJK210-GFP vector. This construct was then transformed into a *ura4-D18 S. pombe* strain. Ura*^+^* transformants were then isolated and subjected to colony PCR to identify clones in which the construct had integrated into the genome via homologous recombination.

To create the Lsg1-myc integrant strain, a C-terminal fragment of the *lsg1* gene was PCR amplified from genomic DNA using primers RS8 and RS14 and cloned into the *SmaI* and *KpnI* restriction sites of the pJK210-myc plasmid. The plasmid construct was then transformed to *S. pombe* strain MBY1343. Ura*^+^* transformants were then isolated and subjected to colony PCR using primers RS9 and RS17 to identify clones in which the construct had integrated into the genome via homologous recombination. To create the Lsg1-HA integrant strain, a C-terminal fragment of the *lsg1* gene was PCR amplified from genomic DNA using primers RS9 and RS16 and cloned into the *SmaI* and *EcoRI* restriction sites of the pJK210-HA plasmid. The plasmid construct was then transformed to *S. pombe* strain MBY1343. Ura*^+^* transformants were then isolated and subjected to colony PCR using primers RS9 and RS16 to identify clones in which the construct had integrated into the genome via homologous recombination.

### Fluorescence Microscopy

*S. pombe* cells expressing Lsg1-GFP fusions, were fixed using ethanol fixation [41] and stored in PBS pH 7.4. To observe nuclei and cell wall/septa material, cells were mixed with 0.02 µg/µL 4’6,-diamidino-2-phenylindole (DAPI) and 1 µg/µL aniline blue. Fluorescent images were obtained using Zeiss Axioskop 2 microscope driven by ImageJ 1.41 software (National Institutes of Health) and Scion CFW Monochrome CCD Firewire Camera (Scion Corporation, Frederick Maryland) using DAPI and GFP filter sets. Gray level linescan measurements were performed using the “plot profile” function of ImageJ. Mean gray level measurements were performed using the “threshold” and “analyze particles” functions of ImageJ.

### Biochemical and Immunological Methods

Cells of the indicated genotype were grown up to the mid-log phase at 30°C, collected by centrifugation, and resuspended in STOP buffer (10 mM Tris-HCl pH 8.0, 150 mM NaCl, 50 mM NaF, 10 mM EDTA, 1 mM NaN_3_). Cell pellets were stored at -80°C up to a maximum of 6 months. Cell pellets were subsequently thawed, and lysed using vortexing with glass beads in extraction buffer (1% IGEPAL CA630 (tetr-Octylphenoxy polyethanol), 150 mM NaCl, 50 mM Tris-HCl pH 8.0, 2 mM EDTA, 1 mM PMSF (phenylmethanesulphonylfluoride), 2 mM Benzamidine, 50 mM NaF, 0.1 mM Na_3_VO_4_, 50 mM B-glycerophosphate, 15 mM p-nitrophenyl phosphate, ¼ Tablet Sigma Protease Inhibitors). Twenty micrograms of total cell extracts were subjected to SDS-PAGE, transferred to PVDF membranes and immunoblotted using one of the following three antibodies: 8WG16 (Sigma), H14 (Sigma) or H5 (Sigma). 8WG16 and H14 were used at 1∶5000 dilutions and H5 was used at a dilution of 1∶1000. Peroxidase conjugated anti-mouse IgG (Sigma) at a dilution of 1∶10000 was used against 8WG16 whereas peroxidase conjugated anti-mouse IgM (Sigma) at a dilution of 1∶10000 was used to recognize H5 and H14 antibodies. In co-immunoprecipitation experiments 2 mg of cell extracts obtained as described above were immunoprecipitated following the manufacturer’s protocol using Protein G Dynabeads (Invitrogen).

### Global Gene Expression Analysis

Strains bearing the *rpb1-12XCTD* and *rpb1-12XS2A* mutations were grown to mid-log phase and treated with 0.5 µM LatA or DMSO for 3 hours at 30°C. Total RNA extraction was performed using the RiboPure Yeast Kit (Ambion) according to the manufacturer’s instructions. Fifteen micrograms of total RNA was processed into labelled cRNA and hybridized to GeneChip Yeast Genome 2.0 Arrays (Affymetrix) by the London Regional Genomic Centre according to the Affymetrix Gene Profiling Reagents User Guide (P/N 702749 Rev. 1) available from the Affymetrix website (http://www.affymetrix.com/support/technical/manuals.affx). Three biological replicates were performed. Global gene expression data was subsequently obtained from the London Regional Genomic Center as CEL computer files containing the raw hybridization signal intensities. Analysis was performed using the Genespring GX 10.0.1 (Agilent Technologies Inc). The CEL files were first normalized with RMA (robust multi-array analysis) algorithm. RMA uses PM (perfect match) probes from the data and corrects the background by fitting a model that is the addition of an exponentially distributed signal and a normally dispersed background [42]. The normalization procedure is helpful in eliminating non-biological sources of variation across individual samples acquired form, for example, differences in hybridization efficiency that varies in different genechips. This generated hybridization intensity data for all twelve samples that were used for further analysis. For quality control purposes Affymetrix includes probes for the *bioB*, *bioC*, *bioD* and *cre* genes on all genechips. During the hybridization protocol different concentrations (1.5, 5, 25, and 100 pmoles) of *bioB*, *bioC*, *bioD* and *cre* transcripts respectively, are “spiked” into the hybridization cocktail in order to assay for hybridization efficiency. As expected, when the data for these genes were inspected, it could be observed that the *cre* gene showed the highest strength of hybridization, followed by *bioD*, *bioC*, and *bioB*. This indicated that the hybridizations had been performed successfully, and that the data could be confidently used in further statistical analysis. The data were grouped under two different categories; genotype, representing *rpb1-12XWTCTD* or *rpb1-12XS2ACTD,* and drug, representing LatA or DMSO treatments. Based on this, all of the data could be classified as 1) *rpb1-12XWTCTD,* DMSO treated, 2) *rpb1-12XWTCTD,* LatA treated, 3) *rpb1-12XS2ACTD*, DMSO treated, and 4) *rpb1-12XS2ACTD*, LatA treated. To identify differentially regulated genes, data for these genes were inspected and data were filtered using Volcano plot analysis employing the Benjamin-Hochberg multiple testing correction. Genes showing statistically significant differences (p-value<0.05) and fold changes greater than 1.5 were identified as differentially regulated. Microarray experiments were performed in compliance with MIAME guidelines. Data has been deposited into the GEO database (Accession Number GSE31369).

## Supporting Information

Figure S1 **Bioinformatic analysis of the** ***S. pombe lsg1*** **gene. (A)** Domain structure of the predicted Lsg1p, Lsc1p and Lsk1p proteins as determined using Prosite. Note that Lsg1p contains a conserved CTK3 superfamily domain at its N-terminus (PFAM12243). Red arrow in Lsk1p indicates the proton acceptor (Asp-399) within the serine/threonine protein kinase active site signature (residues 395-407). **(B)** Reconstructed gene tree of fungal orthogroup OG4121 (Fungal Orthogroups Repository). **(C)** ClustalW protein alignment of members of fungal orthogroup OG4121. *Schizosaccharomyces pombe*, Spom; *Saccharomyces cerevisiae*, Scer; *Saccharomyces mikatae,* Smik; *Saccharomyces paradoxus*, Spar; *Saccharomyces bayanus*, Sbay; *Saccharomyces castellii*, Scas; *Kluyveromyces lactis*, Klac; *Candida glabrata*, Cglab; *Kluyveromyces waltii*, Kwal; *Saccharomyces kluyveri*, Sklu; *Schizosaccharomyces octosporus*, Soct; *Debaryomyces hansenii*, Dhan; *Candida guilliermondii*, Cgui; *Candida lusitaniae*, Clus; *Candida albicans*, Calb; *Candida parapsilosis*, Cpar; *Lodderomyces elongiosporus*, Lelo; *Ashbya gossypii*, Agos; *Schizosaccharomyces japonicus*, Sjap. Lsg1p is marked with a red star.
(TIF)

Figure S2 ***rpb1-12XS2ACTD*** **mutants do not exhibit a generalized defect in mounting a proper transcriptional response to stress. (A)** Volcano plot analysis of the expression of the genes annotated by the Gene Ontology Consortium as having role in the cellular response to stress. Plots compare *rpb1-12XCTD* vs. *rpb1-12XS2ACTD* strains treated with DMSO (top) or LatA (bottom). Horizontal green line represents p-value of 0.05. Vertical green lines represent threshold for a 1.5 fold change in expression. **(B)** Scatter plots comparing genes (squares) annotated by GO as having a cellular response to stress in *rpb1-12XCTD* and *rpb1-12XS2ACTD* strains in the presence of DMSO (top) or LatA (bottom). Diagonal green lines represent the threshold for a 1.5 fold change in expression. Color of squares indicates the level of expression of that gene in DMSO treated *rpb1-12XCTD* cells (top) or LatA treated *rpb1-12XCTD* cells.
(TIF)

Figure S3 **A sub-set of genes differentially regulated in** ***rpb1-12XS2ACTD*** **strains are part of the transcriptional program initiated upon entry into meiosis.** Using data [34] freely available from the Bahler website (http://www.bahlerlab.info/projects/sexualdifferentiation/meiosis/), the expression levels of the indicated genes were plotted versus time after meiotic induction. Data is presented as the ratio between expression level at the indicated times and expression level in vegetative cells. Genes were grouped according to the level and timing of induction/repression.
(TIF)

Table S1 **Summary statistics of log_2_ normalized intensity values.**
(DOCX)

Table S2 **Strains used in this study.**
(DOCX)

Table S3 **Oligos used in this study.**
(DOCX)

File S1 **log_2_ Normalized Intensity Values.**
(XLSX)
