# Supplementary figures and images for: Global Gene Expression Analysis of Fission Yeast Mutants Impaired in Ser-2 Phosphorylation of the RNA Pol II Carboxy Terminal Domain
Source: PLoS One. 2011 Sep 12;6(9):e24694. doi: 10.1371/journal.pone.0024694 (PMC3171476; doi:10.1371/journal.pone.0024694)

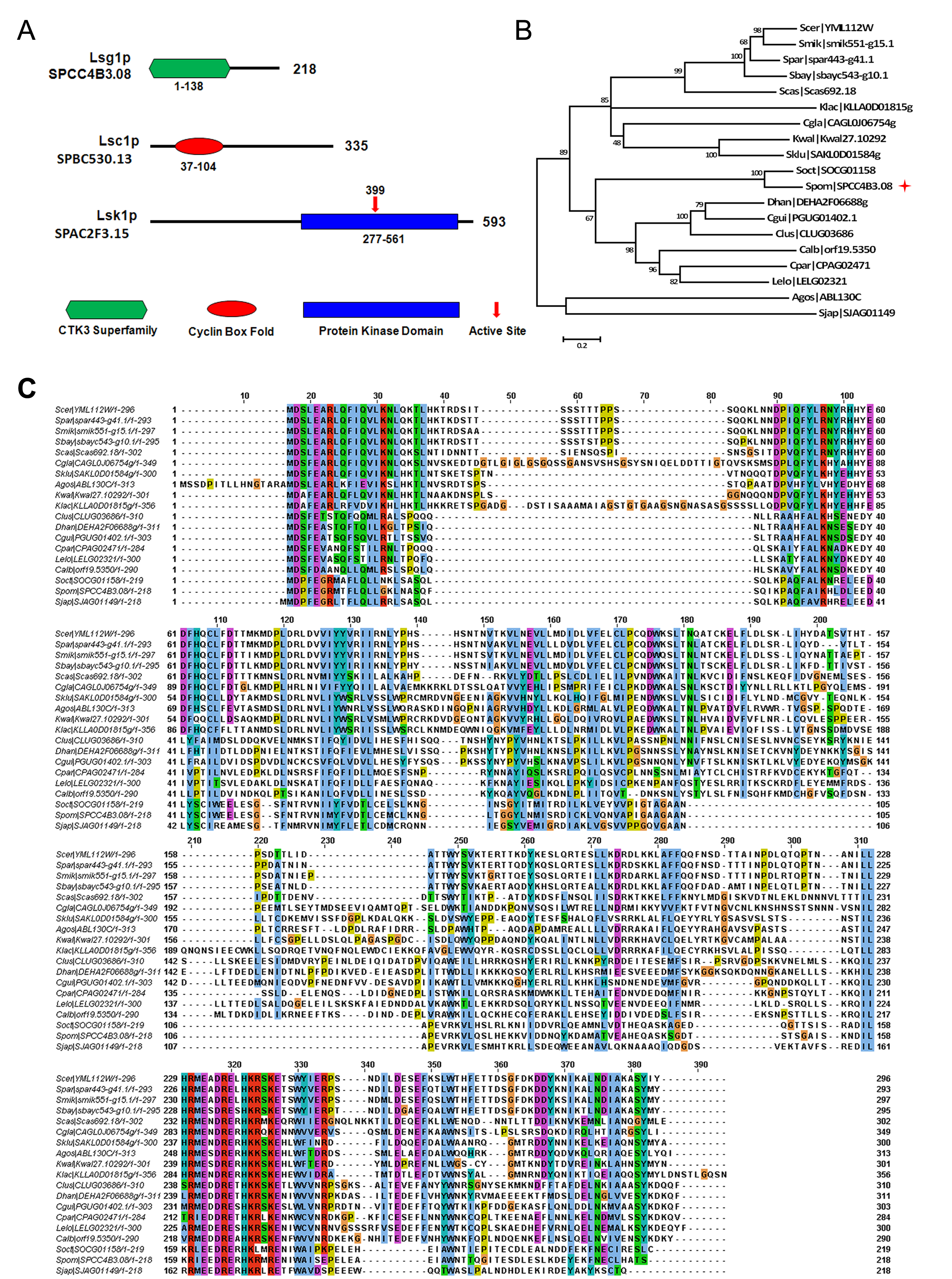

Supplement: Figure S1 — Bioinformatic analysis of the S. pombe lsg1 gene. (A) Domain structure of the predicted Lsg1p, Lsc1p and Lsk1p proteins as determined using Prosite. Note that Lsg1p contains a conserved CTK3 superfamily domain at its N-terminus (PFAM12243). Red arrow in Lsk1p indicates the proton acceptor (Asp-399) within the serine/threonine protein kinase active site signature (residues 395-407). (B) Reconstructed gene tree of fungal orthogroup OG4121 (Fungal Orthogroups Repository). (C) ClustalW protein alignment of members of fungal orthogroup OG4121. Schizosaccharomyces pombe, Spom; Saccharomyces cerevisiae, Scer; Saccharomyces mikatae, Smik; Saccharomyces paradoxus, Spar; Saccharomyces bayanus, Sbay; Saccharomyces castellii, Scas; Kluyveromyces lactis, Klac; Candida glabrata, Cglab; Kluyveromyces waltii, Kwal; Saccharomyces kluyveri, Sklu; Schizosaccharomyces octosporus, Soct; Debaryomyces hansenii, Dhan; Candida guilliermondii, Cgui; Candida lusitaniae, Clus; Candida albicans, Calb; Candida parapsilosis, Cpar; Lodderomyces elongiosporus, Lelo; Ashbya gossypii, Agos; Schizosaccharomyces japonicus, Sjap. Lsg1p is marked with a red star. (TIF) [file pone.0024694.s001.tif]

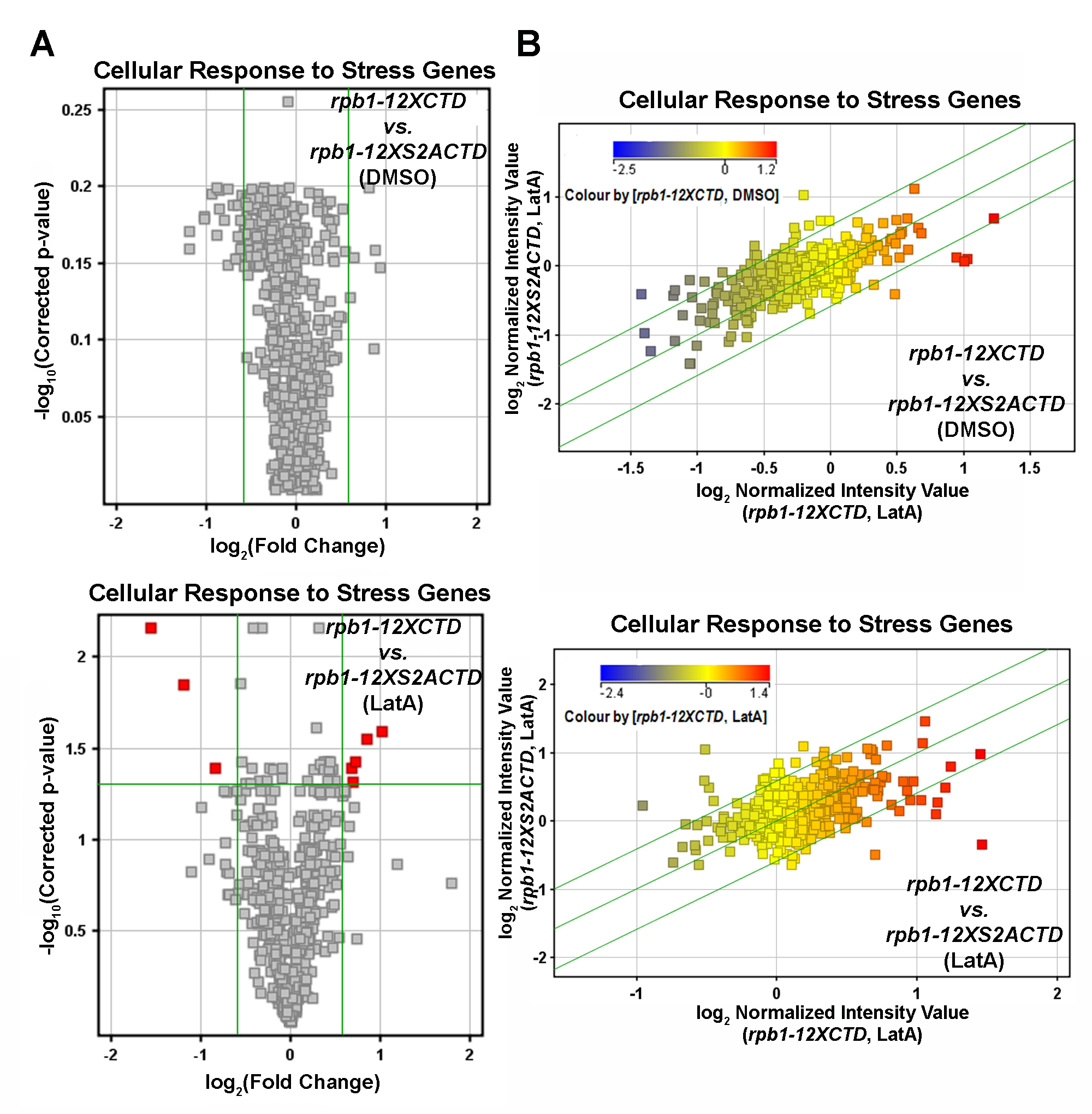

Supplement: Figure S2 — rpb1-12XS2ACTD mutants do not exhibit a generalized defect in mounting a proper transcriptional response to stress. (A) Volcano plot analysis of the expression of the genes annotated by the Gene Ontology Consortium as having role in the cellular response to stress. Plots compare rpb1-12XCTD vs. rpb1-12XS2ACTD strains treated with DMSO (top) or LatA (bottom). Horizontal green line represents p-value of 0.05. Vertical green lines represent threshold for a 1.5 fold change in expression. (B) Scatter plots comparing genes (squares) annotated by GO as having a cellular response to stress in rpb1-12XCTD and rpb1-12XS2ACTD strains in the presence of DMSO (top) or LatA (bottom). Diagonal green lines represent the threshold for a 1.5 fold change in expression. Color of squares indicates the level of expression of that gene in DMSO treated rpb1-12XCTD cells (top) or LatA treated rpb1-12XCTD cells. (TIF) [file pone.0024694.s002.tif]

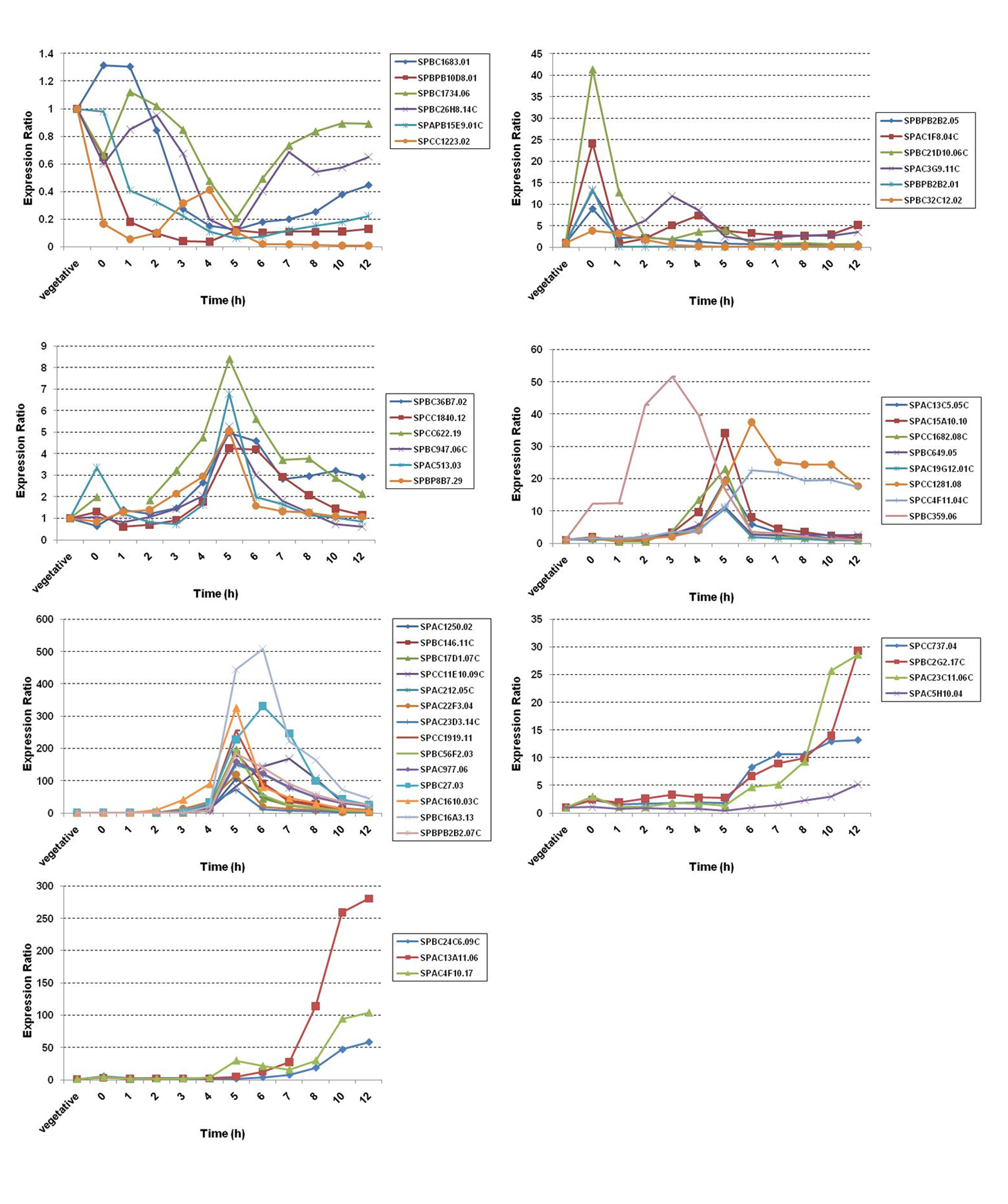

Supplement: Figure S3 — A sub-set of genes differentially regulated in rpb1-12XS2ACTD strains are part of the transcriptional program initiated upon entry into meiosis. Using data [34] freely available from the Bahler website (http://www.bahlerlab.info/projects/sexualdifferentiation/meiosis/), the expression levels of the indicated genes were plotted versus time after meiotic induction. Data is presented as the ratio between expression level at the indicated times and expression level in vegetative cells. Genes were grouped according to the level and timing of induction/repression. (TIF) [file pone.0024694.s003.tif]
