# Supplementary material for: Global Gene Expression Analysis of Fission Yeast Mutants Impaired in Ser-2 Phosphorylation of the RNA Pol II Carboxy Terminal Domain
Source: PLoS One. 2011 Sep 12;6(9):e24694. doi: 10.1371/journal.pone.0024694 (PMC3171476; doi:10.1371/journal.pone.0024694)
Supplement: Table S1 — Summary statistics of log2 normalized intensity values. (DOCX) [file pone.0024694.s004.docx]

| **Table S1:** Summary statistics of log_2_ normalized intensity values. | | | | |
| --- | --- | --- | --- | --- |
|  | ***rpb1-12XCTD*, DMSO** | ***rpb1-12XS2ACTD*, DMSO** | ***rpb1-12XCTD*, LatA** | ***rpb1-12XS2ACTD*, LatA** |
| **Observations** | 4875 | 4875 | 4875 | 4875 |
| **Minimum** | -2.92 | -1.44 | -2.87 | -1.00 |
| **Maximum** | 1.62 | 2.55 | 1.87 | 4.49 |
| **Range** | 4.54 | 3.98 | 4.74 | 5.49 |
| **Mean** | -0.08 | 0.01 | 0.05 | 0.00 |
| **Standard Deviation** | 0.28 | 0.25 | 0.22 | 0.26 |
| **5^th^ Percentile** | -0.52 | -0.35 | -0.24 | -0.39 |
| **10^th^ Percentile** | -0.39 | -0.24 | -0.15 | -0.27 |
| **25^th^ Percentile** | -0.22 | -0.11 | -0.04 | -0.12 |
| **50^th^ Percentile** | -0.06 | 0.00 | 0.03 | 0.00 |
| **75^th^ Percentile** | 0.07 | 0.12 | 0.14 | 0.12 |
| **90^th^ Percentile** | 0.22 | 0.28 | 0.30 | 0.27 |
| **95^th^ Percentile** | 0.32 | 0.40 | 0.41 | 0.38 |
