# Supplementary material for: Global Gene Expression Analysis of Fission Yeast Mutants Impaired in Ser-2 Phosphorylation of the RNA Pol II Carboxy Terminal Domain
Source: PLoS One. 2011 Sep 12;6(9):e24694. doi: 10.1371/journal.pone.0024694 (PMC3171476; doi:10.1371/journal.pone.0024694)
Supplement: Table S2 — Strains used in this study. (DOCX) [file pone.0024694.s005.docx]

| **Table S2:** Strains used in this study. | | |
| --- | --- | --- |
| **Strains** | **Relevant Genotype** | **Source** |
| **RS1** | *lsg1GFP::ura4 ura4-D18 h^+^* | This Study |
| **RS2** | *lsg1::ura4 ura4-D18 h^+^* | This Study |
| **RS9** | *lsg1GFP::ura4 lsk1::ura4 ura4-D18* | This Study |
| **RS36** | *cdc16-116 lsg1::ura4 ura4-D18* | This Study |
| **RS40** | *lsg1myc::ura4 ura4-D18 h^+^* | This Study |
| **RS43** | *lsg1HA::ura4 ura4-D18 h^+^* | This Study |
| **RS46** | *ura4-D18 leu-32 ade6-216 h^+^* | This Study |
| **RS47** | *ura4-D18 leu-32 ade6-216 h^-^* | This Study |
| **RS50** | *lsg1myc::ura4 lsk1HA::ura4 ura4-D18* | This Study |
| **RS56** | *lsg1myc::ura4 lsc1HA::ura4 ura4-D18* | This Study |
| **JK9** | *clp1::ura4 ura4-D18 leu+ h^-^* | JK Collection |
| **JK29** | *lsk1::ura4 ura4-D18 leu1-32 h^-^* | JK Collection |
| **JK327** | *lsc1HA::ura4 ura4-D18 leu1-32 h^-^* | JK Collection |
| **JK328** | *lsk1HA::ura4 ura4-D18 leu1-32 h^-^* | JK Collection |
| **JK366** | *ura4-D18/ura4-D18 leu1-31/leu1-32 ade6-210/ade6-216 h^-^/h^+^* | JK Collection |
| **JK375** | *rpb1-WTCTDX12::ura4 ura4-D18 leu1-32 h^-^* | JK Collection |
| **JK401** | *rpb1-S2ACTDX12::ura4 ura4-D18 leu1-32 h^-^* | JK Collection |
| **JK469** | *aip1::kanMX4 ura4-D18 leu1-32 ade6-M210 h^+^* | JK Collection |
| **JK532** | *aip1::kanMX4 clp1::ura4 ura4-D18* | JK Collection |
| **JK533** | *aip1::kanMX4 lsk1::ura4 ura4-D18* | JK Collection |
| **MBY1343** | *ura4-D18 h^+^* | JK Collection |
| **MBY2432** | *cdc16-116 ura4- leu1-32 ade6-216 M210 h^-^* | JK Collection |
