# Supplementary material for: Global Gene Expression Analysis of Fission Yeast Mutants Impaired in Ser-2 Phosphorylation of the RNA Pol II Carboxy Terminal Domain
Source: PLoS One. 2011 Sep 12;6(9):e24694. doi: 10.1371/journal.pone.0024694 (PMC3171476; doi:10.1371/journal.pone.0024694)
Supplement: Table S3 — Oligos used in this study. (DOCX) [file pone.0024694.s006.docx]

| **Table S3:** Oligos used in this study. | |
| --- | --- |
| **Oligo** | **Sequence** |
| **RS1** | 5'-GGGGGGGTACCTTCATTTCTATGCTTTTCCG-3' |
| **RS2** | 5'-GGGGGCTCGAGGCGAAAACATAGAGTGTAACC-3' |
| **RS3** | 5'-GGGGGTCTAGAGTAATTGCGAGTGCAACAAC-3' |
| **RS4** | 5'-GGGGGGAGCTCCATGCTAACAGTTTGAAGACC-3' |
| **RS5** | 5'-GGGGGCATATGGATCCATTTGAGGGC-3' |
| **RS6** | 5'-GGGGGGTCGACTCAGGAAGTTGCATGAAGAC-3' |
| **RS8** | 5'-GGGGGCCCGGGGGAAGTTGCATGAAGACATTC-3' |
| **RS9** | 5'-GCATGAAAAGAAGGTAATCGA-3' |
| **RS10** | 5'-GTGCTCCAAAGGTTTTGAAA-3' |
| **RS11** | 5'-TTGAAAATTTACTATTTCGCTG-3' |
| **RS14** | 5'-GGGGGGGTACCTAGAGCAAGTCAGGTGATACCAGTA-3' |
| **RS16** | 5'-CGCATAGTCAGGAACATCGT-3' |
| **RS17** | 5'-GAGATTAGCTTTTGTTCACCG-3' |
| **ME12** | 5’-cttatcgataccgtcgacct-3’ |
| **ME28** | 5’-cgggggatccactagttct-3’ |
